# Mechanical and Morphological Changes of the Plantar Flexor Musculotendinous Unit in Children with Unilateral Cerebral Palsy Following 12 Weeks of Plyometric Exercise: A Randomized Controlled Trial

**DOI:** 10.3390/children9111604

**Published:** 2022-10-22

**Authors:** Ragab K. Elnaggar, Mohammed S. Alghamdi, Aqeel M. Alenazi, Mshari Alghadier, Mustafa Z. Mahmoud, Abbas Elbakry A. Elsayed, Ismail Abdelfattah M. Hassan, Asmaa A. Abonour

**Affiliations:** 1 Department of Physical Therapy and Health Rehabilitation, College of Applied Medical Sciences, Prince Sattam Bin Abdulaziz University, Al-Kharj 16278, Saudi Arabia; 2 Department of Physical Therapy for Pediatrics, Faculty of Physical Therapy, Cairo University, Giza 12613, Egypt; 3 Department of Physical Therapy, College of Applied Medical Sciences, Umm Al-Qura University, Makkah 21955, Saudi Arabia; 4 Department of Radiology and Medical Imaging, College of Applied Medical Sciences, Prince Sattam Bin Abdulaziz University, Al-Kharj 16278, Saudi Arabia; 5 Pediatric Department, College of Medicine, Prince Sattam Bin Abdulaziz University, Al-Kharj 16278, Saudi Arabia; 6 Pediatric Department, Faculty of Medicine, Alazhar University, Assiut 71524, Egypt; 7 Pediatric and Neonatology Specialist, New Medical Center, Royal hospital, Khalifa City, Abu Dhabi 35233, United Arab Emirates

**Keywords:** cerebral palsy, ultrasonography, isokinetic, muscle architecture, explosive strength training, Achilles tendon

## Abstract

To investigate how plyometric exercise (PLYO-Ex) affects mechanics and morphometrics of the plantar flexor musculotendinous unit in children with unilateral cerebral palsy, 38 participants (aged 10–16 years) were allocated at random to either the PLYO-Ex group (*n* = 19; received 24 sessions of plyometric muscle loading, conducted 2 times a week for 3 months in succession) or the control group (*n* = 19; underwent traditional physical therapy for the same frequency and duration). Measurements were taken pre- and post-intervention. Standard ultrasound imaging was applied to evaluate morphometrics of the gastrocnemius muscle and Achilles tendon unit and an isokinetic dynamometer was used to evaluate maximum voluntary isometric plantar flexors contraction (IVC_max_). With controlling for pre-treatment values, significant post-treatment changes favoring the PLYO-Ex group were observed for morphological (tendon (*p* = 0.003, *η*^2^_p_ = 0.23) length; belly length (*p* = 0.001, *η*^2^_p_ = 0.27); tendon thickness (*p* = 0.035, *η*^2^_p_ = 0.35); muscle thickness (*p* = 0.013, *η*^2^_p_ = 0.17); fascicle length (*p* = 0.009, *η*^2^_p_ = 0.18); pennation angle (*p* = 0.015, *η*^2^_p_ = 0.16)) and mechanical and material properties (IVC_max_ (*p* = 0.009, *η*^2^_p_ = 0.18); tendon’s elongation (*p* = 0.012, *η*^2^_p_ = 0.17), stiffness (*p* = 0.027, *η*^2^_p_ = 0.13); stress (*p* = 0.006, *η*^2^_p_ = 0.20); strain (*p* = 0.004, *η*^2^_p_ = 0.21)). In conclusion, plyometric exercise induces significant adaptations within the musculotendinous unit of the plantar flexors in children with unilateral cerebral palsy. These adaptations could improve muscular efficiency and consequently optimize physical/functional performance.

## 1. Introduction

Cerebral palsy (CP) is a set of neurodevelopmental disorders manifesting in childhood and permanently affects children’s capacity of motor control (i.e., compromise mobility and posture). CP derives its origin from an insult or malformation of a developing brain [1]; nonetheless, it causes consequent alterations to the musculoskeletal system [2,3], such as weakness, limited joint mobility, and increased passive stiffness, thereby reducing motor function in daily living activities [4].

While functional capacity is contingent upon the acting muscle-tendon unit, recent investigations on children with spastic CP placed emphasis on examining the architecture and morphology of the musculotendinous unit of the plantar flexors, being the most affected muscle [5,6]. These investigations delivered evidence on reduced length and volume of the muscle belly, decreased pennation angle, and decreased fascicle length, when compared to the typically developing children [6,7]. In addition, recent works have shown increased length of the Achilles tendon and reduced cross-sectional area [8,9]. Examinations of the mechanical properties have demonstrated increased plantar flexor and ankle stiffness in children with spastic CP [4,10]. Collectively, these changes could contribute to muscle weakness that is known to be a significant impairment factor limiting the mobility of children with CP [11,12].

Many exercise-based interventional approaches have been used to enhance motor function [13]. Plyometric exercise (PLYO-Ex) is an explosive strength training strategy that causes muscles to quickly reach their maximum force [14]. Prior investigations on healthy individuals indicated that such a training paradigm is associated with certain neuromuscular, mechanical, and morphological adaptations. During PLYO-Ex, a quick repeated cycle of eccentric and strong concentric contraction is generated, which changes the stretch reflex excitability (i.e., heightens the myotatic reflex), thus, contributing to increased motor unit recruitment, force/torque development rate, and musculotendinous strength [15,16,17]. The available evidence further suggested that PLYO-Ex induces some changes in the muscle’s geometrical and mechanical properties; these included, enhanced contractility, reduced contraction time, increased single muscle fiber size and whole muscle volume, increased fascicle length, and decreased fascicle angle [18].

PLYO-Ex has been found to help children with CP gain strength and function [19,20,21,22]. Changes at the muscle level are thought to contribute to such gains; this, however, has not been fully examined. According to a recent review [23], there is insufficient research on how strength training affects the morphology and architecture of skeletal muscles. Lee et al. [24], and Williams et al. [25], claimed that strength training can increase gastrocnemius muscle thickness and volume and enhance fascicle pennation angle. However, there is still a lack of information on some intervention-related changes at the muscle level, such as fascicle length. Moreover, information is also lacking regarding the role of strength training for the mechanical/material characteristics of the Achilles tendon (e.g., tendon stiffness, stress, and strain). Zhao et al. [26] studied the integrated effect of active movement training and passive stretching. Nevertheless, the effects of its components could not be distinguished due to the use of combined training.

The PLYO-Ex is an alternative strength training method that is carried out with faster and more functional movement speed compared to the most frequently-used strength training approach (i.e., progressive resistance training). This strategy has been shown to help children with CP in developing strength and function more effectively than the traditional methods employing slow repetitive single-joint movements [19,20,27,28]. This also supports the potential that PLYO-Ex may evoke mechanical and morphological modifications at the muscle level and enhance physical ability. To date, scant attention has been paid to studying the effect of PLYO-Ex training at the muscular level in children with CP. Therefore, this study was carried out on a convenience sample of children with unilateral CP to explore the mechanical and morphological characteristics of the plantar flexor muscle-tendon after 3 months of PLYO-Ex.

## 2. Materials and Methods

### 2.1. Experimental Design and Ethics

From November 2020 to December 2021, we performed a two-arm, single-blind, randomized controlled experiment at the Laboratories and Physical Therapy Center at PSAU, Al-Kharj, KSA. The data collectors were blinded to the treatment conditions. The procedures were conducted in keeping with the Helsinki Declaration’s ethical guidelines of 1975 and its recent iteration in 2013. The Ethics Review Board at PSAU approved the study protocol (Protocol No: RHPT/0020/0059) on March 1, 2020. Participants/guardians were informed of study procedures, benefits, and potential risks and were asked to sign a written consent form in before data collection. The experimental protocol was documented and registered at ClinicalTrial.gov (NCT05301738).

### 2.2. Participants

Thirty-eight children were recruited from the PSAU’s Physical Therapy Center and two referral hospitals, Al-Kharj, KSA. Inclusion criteria were a definitive unilateral CP diagnosis [29], age from 10 to 16 years, spasticity level 1 or 1+ [30], motor function level I or II [31], absence of clinically-relevant plantar flexors’ contracture–that is, with extended knee, the maximal dorsiflexion RoM was ≥5 degrees [5], and sufficient mental function (of note, intellectual functionality was determined if the children could properly perceive, understand, and follow directions during the preliminary screening session, attend typical classrooms where they are able to focus, communicate, and participate in traditional learning, and their records, if already containing pertinent information, revealed that they possessed a normal mental capacity). Exclusion criteria were non-reducible lower extremity deformities, botulinum toxin-A injection to plantar flexors within the last 6 months, orthopedic and/or neuromuscular surgery through the previous 12 months, and cardiac or pulmonary disorders.

#### 2.2.1. Assignment Procedure

A non-participating independent researcher assigned eligible participants randomly into either the PLYO-Ex group (n = 16; to take part in a plyometric training regimen) or the control group (n = 16; to receive the traditional physical therapy). To ensure equal numbers were allocated to each group, Permuted blocks of various sizes were used for randomization. A consecutively numbered sequence of sealed, opaque envelopes (containing random-generator selected numbers) was created in each block. Once a participant was formally entered, the researcher unsealed each next envelope in sequence. 

#### 2.2.2. Sample Size Determination

In order to figure out the appropriate sample size, we performed an a priori power analysis via PASS software, v14.0.15 (NCSS, Kaysville, UT, USA) relying on estimates of the means (μ1 = 1.18, μ2 = 1.02) and a common with-group standard deviation (SD = 0.19) for the normalized plantar flexors’ muscle-tendon length, which were collected from a preliminary study. The magnitude of the difference between the means has been represented by their SD, which was 0.08. In a one-way ANCOVA study with a covariate R^2^ value of 0.52, a total sample size of 32 participants (a group-sample of 16) was needed to achieve a power of 91% to identify inequalities between the means against the alternative of equal means using an F test with a 5% risk of rejecting the null hypothesis when it is true. To compensate for some dropout rates, we recruited 38 participants (19 per group). 

### 2.3. Measurements

#### 2.3.1. Baseline Measurements

Participants’ height (H; in meters) and weight (W; in kilogram) were measured using a wall-mounted-stadiometer and a calibrated balance beam scale, respectively. The height was rounded to the nearest 0.1 cm and weight was recorded within 0.1 kg precision. The body mass index was then calculated through the commonly accepted formula [BMI = W (Kg)/H (m^2^)]. The leg length was assessed using a direct clinical method (i.e., with a flexible tape). The whole leg length was assessed while participants were assuming a supine position with the pelvis leveled and the body fully extended and was determined as the distance between the anterior superior iliac spine and the medial malleoli of the ankle. For the purpose of the present study, the lower leg length was also measured from the prone lying position with the knees flexed to 90° as the distance between the medial articulation of the knee joint to the most distal border of the medial malleoli of the ankle to be used in subsequent analyses.

#### 2.3.2. Plantar Flexors’ Morphological Properties

A standard high-resolution ultrasound imaging system (Logiq 500, GE Medical Systems, Milwaukee, Wisconsin) equipped with a linear-array transducer (10-cm; 10 MHz; 74-mm depth) was used for investigation of the plantar flexors and the muscle-tendon unit’s morphological features. Examinations were performed on the paretic side while children were assuming a prone laying position with their feet dangling from the examining table’s edge and their knees in full extension. Before ultrasound scanning, the resting angle of the ankle joint (i.e., foot in a relaxed position with no additional stress applied by the examiner) was measured using a standard goniometer. From the resting position, the moment arm of the AT was individually measured by a metric tape as the distance between the AT action line and the lateral malleolus [32]. The length of the gastrocnemius muscle belly/muscle-tendon unit and AT were measured as detailed in a previous study [33] and were further normalized to the length of the lower leg. Fascicle length (distance between the superficial and deep aponeurosis where the insertion is located) [8], pennation angle (the fascicle’s angle with the deep aponeurosis) [26], and gastrocnemius muscle thickness (thickness between the superficial and deep aponeurosis, measured in perpendicular distance) were also measured [34]. All participants were examined by the same investigator pre- and post-treatment.

#### 2.3.3. Plantar Flexors’ Mechanical Properties

The maximal isometric voluntary contraction (IVC_max_) and passive range of motion (RoM) of the ankle joint were measured using an isokinetic dynamometer (Humac Norm CSMI 2009, Stoughton, MA, USA). Participants were seated with their leg extended to the furthest extent in line with their thighs, Velcro straps were applied to secure and hold the hip and shoulder in position, the dynamometer’s rotating axis was adjusted at the ankle joint center (axis through the lateral malleolus), and the foot was securely strapped to a foot-plate [5]. To measure ankle angle and RoM, the ankle joint was manually moved by the examiner into maximum dorsi- and plantar flexion until participants reported discomfort. To measure IVC_max_ of plantar flexors, the ankle joint was rotated passively to dorsiflexion, then, two 5-s IVC_max_ were performed in a neutral ankle position (i.e., a 90-degree angle between the foot and the leg), with a one-minute rest in-between. During measurement, participants were provided with consistent verbal encouragement and real-time visual feedback via a computer interface that displays the torque development curve. Since the study’s participants were children with unilateral CP who are relatively weaker compared to their healthy counterparts and were anticipated to have difficulty executing controlled ramp contractions, all participants were urged to achieve IVC_max_ as swiftly as they could (i.e., with the highest possible rate of force generation) [35]. The testing trials were preceded by two submaximal voluntary contractions to familiarize participants with the testing procedures. The highest torque value of both trials was documented and processed for further analysis [5]. 

During the IVC_max_ assessment, the muscle-tendon elongation (i.e., gastrocnemius muscle-tendon junction displacement) was examined concurrently via longitudinal ultrasound images captured at a rate of 25 Hz (72-mm depth; 10 MHz) as previously described [5,36]. The ultrasound videos and torque/angle data were synchronized by means of a trigger signal. Then, a frame-by-frame tracking of the muscle-tendon junction from the ultrasound video was done to find out how far the muscle and the tendon have displaced [37]. A low-pass, second-order, zero-lag Butterworth filter was used to filter muscle elongation data, with a cut-off frequency of 3 Hz. Thereafter, the length changes of the musculotendinous unit during passive movement were estimated as a function of the ankle joint angle [38]. The Achilles tendon (AT) elongation was defined as the difference between the ultrasound-measured changes in muscle length and the length changes computed for the musculotendinous unit. 

The absolute AT force was calculated through the division of the ankle torque by the moment arm of the AT over a range from the resting angle of the ankle joint to the measured maximum dorsiflexion. The active AT stiffness and ankle joint stiffness were determined through linear regression of the tendon force and corresponding length changes, as well as ankle torque and related ankle angle changes, respectively. The regression analysis was performed over a range between 50% and 90% of peak torque and force. Furthermore, the AT strain (tendon elongation/initial length) and stress (passive AT tendon force/tendon’s cross-sectional area) were calculated to determine Young’s modulus [5]. To adjust for assumed between-group torque disparities, parameter computations were performed for a common torque interval (between zero and the least toque achieved through pre-/post-treatment measurements). Therefore, regardless of any strength gain, a direct comparison of mechanical properties can be made.

### 2.4. Interventions

#### 2.4.1. Plyometric Exercises

Participants in the PLYO-Ex group received 24 sessions of plyometric exercises over 3 consecutive months. Training sessions were repeated twice per week (with rest intervals of at least 48 h) under a pediatric physical therapist’s supervision. Every session was approximately 45 min and included warm-up (5 min), workout (~35 min), and cool-down (5 min) phases. In the warm-up and cool-down phases, children performed dynamic/functional stretching exercises and low-level bicycle or treadmill exercises as each child preferred. The plyometric workout was implemented as described previously [20]—a detailed characterization of plyometric exercises and their progression is demonstrated in Supplementary Table S1. In the plyometric workout phase, children performed 10 lower limb-directed exercises in 2 training paradigms (i.e., vertical/horizontal exercises). The number of sets/repetitions was increased across three exercise blocks (each one lasted a month) to achieve training progression. The training was undertaken in keeping with to the National Strength and Conditioning Association guidelines [39] and the American Academy of Pediatrics safety standards [40]. The children’s performance was preliminarily assessed in a group of six children to figure out the training volume (number of sets/repetition) they should begin within the first block. Children were provided with a pre-training session to teach them about the proper implementation of the PLYO-Ex and were continuously reminded about that during training. All plyometric activities were undertaken on a rubber ground with the participants putting on sports clothing and footwear.

#### 2.4.2. Traditional Physical Therapy

The control participants underwent a traditional physical therapy program, 45-min/session, 2 times a week over a course of 3 consecutive months, totaling 24 sessions. The program was customized to fit each participant’s demands and it was run by a physical therapist with more than 10 years of expertise in pediatric rehabilitation. The main concerns of the program were: overcoming physical limitations, optimizing motor skills, increasing flexibility and strength, enhancing balance capabilities, correcting abnormal walking patterns, and minimizing compensatory movement patterns. The program was designed to subject the plantar flexors to as much equivalent training load and volume as in the PLYO-Ex program. While plyometric-related activities have an explosive and dynamic nature that is associated with enhanced motor unit recruitment (on account of the heightening of the myotatic reflex of the plantar flexor muscles), rapid change in the muscles’ length and tension, repetitive and strong activation of the mechanical receptors, and a wide range of balance difficulties that induce neuromuscular adjustments in the plantar flexor muscle-tendon unit [18,20,27], the traditional physical therapy program was crafted to incorporate exercises that might provide the plantar flexor muscle-tendon unit with comparable stimuli. The program was made up of a variety of flexibility exercises (functional stretches), functional strength training based on progressive resistance exercises, targeted muscle activation exercises, postural re-education exercises, advanced balance exercises, and gait training.

### 2.5. Data Analysis

Data computations were completed through NCSS statistical software, v11.0.13 (NCSS Statistical Software©, Kaysville, UT, USA). The hypothesis that data were sampled from a normal distribution was checked through the D’Agostino Omnibus test of normality. The baseline homogeneity was analyzed using an unpaired *t*-test and Fisher’s exact test, respectively, for continuous and categorical variables. To preserve the prognostic balance afforded by randomization, the intention-to-treat analysis was utilized for comparing groups. Missing data were handled using a partial imputation method, where the pre-treatment observations of participants who deviated from the study were carried forward to make up for missed follow-up data. The post-treatment differences between groups were computed using the One-way ANCOVA test. The pre-treatment scores of each dependent variable were used as covariates. In cases where the ANCOVA test indicated a significant between-group difference, effect size (the magnitude of the difference) was computed employing the Partial eta-squared (*η*^2^_p_) equation. For all analyses, the alpha level was maintained at 0.05%. Partial eta-squared values are interpreted as follows: *η*^2^_p_ = 0.01 denotes a “small” effect, *η*^2^_p_ = 0.06 implies a “medium” effect, and *η*^2^_p_ = 0.14 signifies a “large” effect size [41].

## 3. Results

### 3.1. Enrollment and Retention

Initially, 79 children were screened. Thirty-eight of them satisfied the eligibility criteria and proceeded to the randomization phase. Three participants (7.9%) were lost prematurely (i.e., one child from PLYO-Ex group (5.3%) and two from the control group (10.5%)), where they either failed to finish the prescribed intervention or to attend the follow-up measurements due to scheduling issues or a move out of the working area. However, consistent with the intention-to-treat principle, their data were substituted and included in the statistical analysis.

### 3.2. Baseline Characteristics

There was no noticeable difference between the PLYO-Ex and control groups concerning age, gender, and anthropometry (height, weight, and BMI) at the baseline. Additionally, there was no discernible difference between the two groups in terms of the clinical characteristics (i.e., paretic side, motor function level, spasticity grade, lower leg length, and ankle-foot orthosis use) (Table 1). Furthermore, all dependent outcome measures (muscle-tendon morphological properties, RoM and active peak torque, and muscle-tendon mechanical and material properties) in both groups were homogenous as regards the baseline assessment (Table 2).

### 3.3. Between-Group Differences

Per the ANCOVA analysis, the muscle belly length [*F*_(1,35)_ = 13.23, *p* = 0.001, *η*^2^_p_ = 0.27], AT cross-sectional area [*F*_(1,35)_ = 4.83, *p* = 0.035, *η*^2^_p_ = 0.21], fascicle length [*F*_(1,35)_ = 7.69, *p* = 0.009, *η*^2^_p_ = 0.18], pennation angle [*F*_(1,35)_ = 6.61, *p* = 0.015, *η*^2^_p_ = 0.16], and muscle thickness [*F*_(1,35)_ = 6.92, *p* = 0.013, *η*^2^_p_ = 0.17] increased significantly in the PLYO-Ex group in comparison with the control group. Additionally, the AT length [*F*_(1,35)_ = 10.38, *p* = 0.003, *η*^2^_p_ = 0.23] decreased considerably in the PLYO-Ex group relative to the control group. However, changes in muscle-tendon length [*F*_(1,35)_ = 0.97, *p* = 0.33, *η*^2^_p_ = 0.03] were comparable in both groups (Table 3).

In addition, significant post-treatment increases in the ankle RoM [*F*_(1,35)_ = 22.87, *p* < 0.001, *η*^2^_p_ = 0.39], maximum dorsiflexion [*F*_(1,35)_ = 7.04, *p* = 0.012, *η*^2^p = 0.17], maximum plantar flexion [*F*_(1,35)_ = 13.88, *p* = 0.001, *η*^2^_p_ = 0.28], and plantar flexors’ IVC_max_ [*F*_(1,35)_ = 7.74, *p* = 0.009, *η*^2^_p_ = 0.18], and significant decreases in the ankle’s resting angle [*F*_(1,35)_ = 7.24, *p* = 0.011, *η*^2^_p_ = 0.17] were found in the PLYO-Ex group as opposed to the control group (Table 4). 

Furthermore, the post-treatment AT elongation [*F*_(1,35)_ = 7.11, *p* = 0.012, *η*^2^_p_ = 0.17], AT stiffness [*F*_(1,35)_ = 5.32, *p* = 0.027, *η*^2^_p_ = 0.13], AT strain [*F*_(1,35)_ = 9.35, *p* = 0.004, *η*^2^_p_ = 0.21], and AT stress [*F*_(1,35)_ = 8.56, *p* = 0.006, *η*^2^_p_ = 0.20] increased markedly in the PLYO-Ex group in comparison with the control group. However, changes in Young’s modulus [*F*_(1,35)_ = 0.08, *p* = 0.78, *η*^2^_p_ = 0.002] were similar in both groups (Table 5).

## 4. Discussion

This study examined the effect of three-month PLYO-Ex training on the mechanical and morphological characteristics of the plantar flexor musculotendinous unit in a cohort of children with unilateral CP. A particularly notable finding in the present study is that children who underwent PLYO-Ex demonstrated favorable improvements in the majority of the mechanical and morphological characteristics of the plantar flexor musculotendinous unit in comparison with the control group.

As far as we are aware, this is the inaugural study to look into how PLYO-Ex modulates the plantar flexor muscle-tendon unit in children with CP. Evaluation of interventions at the muscular level is crucial especially since prior research has demonstrated that therapies might increase functionality while adversely changing the structural characteristics, which might aggravate the muscular weakness further [42]. Our finding that a majority of the morphological properties changed in favor of the PLYO-Ex training extends the evidence on the positive implications of such a training model for children with unilateral CP [19,20,21,27]. The observed improvement in muscle thickness, cross-sectional area, muscle belly, and tendon length for children in the PLYO-Ex might reflect an appropriate dose-response trend for the training regimen implemented in this study. That is, the training dose (i.e., intensity, volume, and duration) was sufficient to evoke morphological alterations of the musculotendinous structures. These findings seem to be consistent with earlier reports where functional strength training is associated with increased muscle thickness and the cross-sectional area [5,24,25]. The morphological properties of muscles respond to different types of stimuli, such as immobilization, exercises, and injury [43]. The morphological alterations of spastic limb muscles in children with unilateral CP are hypothesized to result from the limited range of mobility and the associated neuromuscular restraint brought on by the brain injury, which in consequence, influence the force-generating capacity of these muscles [6]. So, the favorable morphological changes demonstrated herein may represent an adaptation to the intense mechanical and neurological stimuli that the PLYO-Ex imposed on the plantar flexors.

The IVC_max_ also improved following PLYO-Ex, suggesting improved activation capacity of plantar flexors. The voluntary activation capacity of planter flexors is typically decreased in children with spastic CP [44]. O’Brien and colleagues [44], found similar planter flexors’ voluntary activation capacity between children with spastic CP (motor function levels I–III) and their normal peers. However, they suggested that muscle size rather than voluntary activation ability could be more responsible for weakness in children with CP classified as motor function levels I–III given that strength deficits were not predicted by voluntary activation capacity. We believe that muscle properties in paretic limbs are interrelated and contribute to strength and functional deficits, but the exact proportion is challenging to define. With regards to the ankle joint mobility, our findings that total ankle RoM and the maximum dorsi/plantar flexion improved following PLYO-Ex may illustrate the concurrent impact of PLYO-Ex training on muscle architecture/strength and joint flexibility. Likely, the improved architectural qualities and higher force-producing capability allowed the ankle muscles to work on a wider range of motion. 

Similar to changes in morphological properties, PLYO-Ex led to positive changes in AT mechanical and material properties. Barber and colleagues [4] found that AT slack length is 10% longer in children with spastic CP compared to their healthy counterparts. They suggested that this contributes to the storage and recovery of elastic energy and could act as a compensation strategy for reduced force production. In addition, Kruse and colleagues [5] also reported that AT stiffness in children in motor function levels I-II is more compliant to low forces such as passive stretching. Our observation that changes in Young’s modulus were equivalents in the PLYO-Ex and control groups might be attributed to the great variability in Young’s modulus values between participants in both groups. Overall, changes in mechanical properties suggest that plyometric training contributed to a greater capacity to store and recover elastic strain energy in the AT.

The exact mechanisms whereby the PLYO-Ex caused the plantar flexor muscle-tendon unit to change mechanically and morphologically are not fully understood. However, there are justifiable models (neurophysiological and mechanical) for the explanation of these favorable changes. In PLYO-Ex, a quick, repeated cycle of powerful eccentric and concentric contraction is produced, which alters the stretch reflex excitability (that is, it heightens the myotatic reflex by evoking stronger signals from the muscle spindles) and boosts the recruitment of motor units, the rate at which force and torque are developed, and the reactive strength of the muscle-tendon complex [15,16,17]. From the biomechanical standpoint, the PLYO-Ex enhances the force/torque production by inducing an elastic recoil of the tendon and extending the timespan for developing the force (i.e., stores kinetic energy during the eccentric phase which enhance the contractile force during the ensuing concentric phase) [45]. The enhanced IVCmax in response to PLYO-Ex in the current investigation may thus be adequately justified by these neurophysiological and mechanical adaptations.

Given the relationship between the increasing weakness and morphological and material characteristics of a muscle in children with CP [6,22], it can be inferred that the increased strength/torque development and gain after PLYO-Ex (although not directly measured in the present study but verified previously [19]) could have been associated with changes at the muscular level. It is well documented that PLYO-Ex increases the active muscle range of the length-tension relationship, especially since they take advantage of the quick cyclic muscle action (i.e., stretch-shortening cycle) to effectively generate force in the shortest possible time. Therefore, the conducive changes in morphological properties (i.e., muscle/tendon length, muscle thickness/cross-sectional area, fascicle length, and pennation angle) might have occurred as consequent structural adaptations matching the changes in mechanical properties of the plantar flexor muscle-tendon unit [22]. In addition, with PLYO-Ex, the muscle fibers might have transduced the stress and strain, and, in response, modified the number of sarcomeres in a way that is better adapted to the imposed mechanical challenges [46]. Furthermore, the increase in AT stiffness might be linked to the tendon structure’s adaptability and remodeling at the cellular level in response to the intense and repeated mechanical stimuli during training.

This study has some limitations worthy of mention. Owing to the intense and physically-demanding nature of the PLYO-Ex, this study included only children with unilateral CP (mild cases; motor function level I or II and spasticity grade 1 or 1+) who had less physical impairment than those with other types of CP, which may limit the applicability of the current findings to children with other types of CP and even those with more severe cases of unilateral CP. Therefore, exploring the efficacy of PLYO-Ex in larger samples with representation from all forms of CP is a logical next step of the current work to provide conclusive proof. The limited sample size and lack of the long-term follow-up may also affect the generalizability of the findings. Future research should examine the effect of plyometric exercise in children with CP with longer follow-up times to examine the long-term effects and the retention of improvements. Some outcomes might be subject to measurement errors and variability, although we tried to minimize errors by further training for data collectors. Finally, despite the fact that there were no significant differences between groups concerning the dependent variables at the baseline, data observation ahead of the analysis demonstrated that certain children (who had higher baseline scores) achieved relatively greater positive changes. Therefore, we analyzed the data using analysis of covariance (i.e., baseline scores were factored in the model as covariates) to provide a straightforward comparison and to minimize the possible impact of the baseline scores.

In conclusion, the effect of using plyometric exercise was evident in improving mechanical and morphological outcomes for the planter flexor muscle-tendon unit when compared to traditional physical therapy in people with unilateral cerebral palsy. Future research should investigate the long-term effectiveness of plyometric exercise in this population.

## Figures and Tables

**Table 1:** Baseline characteristics of the participating children in the PLYO-Ex and control group.

Variables	PLYO-Ex Group (*n* = 19)	Control Group (*n* = 19)	*p*-Value
Age, *years*	12.95 ± 1.87	13.53 ± 1.81	0.34 ^a^
Gender (boys/girls), *n* (*%*)	11 (57.9)/8 (42.1)	13 (68.4)/6 (31.6)	0.74 ^b^
Weight, kg	47.53 ± 7.10	50.42 ± 8.22	0.25 ^a^
Height, m	1.49 ± 0.12	1.50 ± 0.13	0.75 ^a^
BMI, kg/m^2^	21.36 ± 1.45	22.25 ± 1.73	0.09 ^a^
Paretic side (RT/LT), *n* (*%*)	4 (21.1)/15 (78.9)	7 (36.8)/12 (63.2)	0.48 ^b^
Spasticity level on MAS (1/1+), *n* (*%*)	10 (52.6)/9 (47.4)	14 (73.7)/5 (26.3)	0.31 ^b^
GMFCS level (I/II), *n* (*%*)	13 (68.4)/6 (31.6)	16 (84.2)/3 (15.8)	0.45 ^b^
Lower leg length, cm	37.39 ± 4.30	38.60 ± 3.09	0.52 ^a^
Use of AFO (yes/no), *n* (*%*)	5 (26.3)/14 (73.7)	8 (42.1)/11 (57.9)	0.49 ^b^
AFO use/day, h/day	4.52 ± 1.87	3.74 ± 1.76	0.19 ^a^
Duration of AFO use, *months*	21.16 ± 6.34	19.26 ± 6.89	0.38 ^a^

Abbreviations: PLYO-Ex: plyometric exercises, BMI: body mass index, RT: right, LT: left, MAS: Modified Ashworth Scale, GMFCS: gross motor function classification system, AFO: ankle foot orthosis. ^a^: unpaired *t*-test, ^b^: Fisher’s exact test.

**Table 2:** Dependent outcome measurements at baseline in the PLYO-Ex and control groups.

Variables	PLYO-Ex Group (*n* = 19)	Control Group (*n* = 19)	*p*-Value
**Muscle-tendon morphological properties**			
Muscle-tendon length, *normalized*	1.04 ± 0.07	1.01 ± 1.06	0.19
AT length, *normalized*	0.53 ± 0.07	0.51 ± 0.05	0.37
Muscle belly-length, *normalized*	0.48 ± 0.04	0.48 ± 0.06	0.81
AT cross-sectional area, mm^2^	45.55 ± 5.70	45.85 ± 4.11	0.85
Fascicle length, mm	38.52 ± 6.21	37.84 ± 4.34	0.69
Pennation angle, *degree*	18.11 ± 3.59	18.21 ± 2.78	0.92
Muscle thickness, mm	14.68 ± 3.73	13.37 ± 2.85	0.23
**RoM and active peak torque**			
Ankle RoM, *degree*	53.47 ± 5.79	52.86 ± 5.10	0.73
Maximum dorsiflexion, *degree*	12.21 ± 4.16	11.95 ± 2.59	0.82
Maximum plantar flexion, *degree*	−41.27 ± 4.26	−40.91 ± 4.15	0.79
Resting angle, *degree*	−22.05 ± 3.79	−21.63 ± 2.81	0.70
IVC_max_, N·m	37.53 ± 9.15	36.59 ± 10.26	0.77
**Muscle-tendon mechanical and material properties**			
AT elongation, mm	3.43 ± 1.46	3.54 ± 0.64	0.78
AT stiffness, N/mm	37.35 ± 17.05	65.27 ± 13.71	0.68
AT strain, %	2.58 ± 0.76	2.52 ± 0.80	0.81
AT stress, N/mm^2^	4.55 ± 1.37	4.31 ± 1.26	0.58
Young’s modulus, N/mm^2^	197.48 ± 96.91	191.35 ± 85.57	0.84

Abbreviations: PLYO-Ex: plyometric exercises, AT: Achilles tendon, RoM: range of motion, IVC_max_: maximum voluntary isometric contraction.

**Table 3:** Post-treatment differences in muscle-tendon morphological properties (normalized to length of the leg) between the study group.

Variables	PLYO-Ex Group (*n* = 19)	Control Group (*n* = 19)	*p*-Value	*η* ^2^_p_
Muscle-tendon length, *normalized*	1.06 ± 0.09	1.02 ± 0.06	0.33	0.03
AT length, *normalized*	0.48 ± 0.06	0.50 ± 0.05	0.003 *	0.23
Muscle belly-length, *normalized*	0.51 ± 0.06	0.49 ± 0.04	0.001 *	0.27
AT cross-sectional area, mm^2^	47.17 ± 6.05	46.61 ± 3.80	0.035 *	0.12
Fascicle length, mm	40.89 ± 5.57	38.42 ± 3.91	0.009 *	0.18
Pennation angle, *degree*	20.26 ± 3.45	19.11 ± 2.38	0.015 *	0.16
Muscle thickness, mm	16.53 ± 3.70	14.11 ± 2.58	0.013 *	0.17

Abbreviations: PLYO-Ex: plyometric exercises, AT: Achilles tendon. * Significant at *p* < 0.05, *η*^2^_p_: partial eta-squared.

**Table 4:** Post-treatment differences in ankle RoM and IVC_max_ between the PLYO-Ex and control group.

Variables	PLYO-Ex Group (*n* = 19)	Control Group (*n* = 19)	*p*-Value	*η* ^2^_p_
Ankle RoM, *degree*	57.86 ± 5.33	54.31 ± 3.59	<0.001 *	0.39
Maximum dorsiflexion, *degree*	14.19 ± 3.92	12.92 ± 2.15	0.012 *	0.17
Maximum plantar flexion, *degree*	−43.67 ± 3.67	−41.39 ± 3.36	0.001 *	0.28
Resting angle, *degree*	−19.95 ± 2.46	−20.84 ± 2.63	0.011 *	0.17
IVC_max_, N·m	43.75 ± 8.13	40.39 ± 8.21	0.009 *	0.18

Abbreviations: PLYO-Ex: plyometric exercises, RoM: range of motion, IVCmax: maximum voluntary isometric contraction. * Significant at *p* < 0.05, *η*^2^_p_: partial eta-squared.

**Table 5:** Post-treatment differences in ankle muscle-tendon mechanical and material properties (calculated at the common peak torque) between the PLYO-Ex and control group.

Variables	PLYO-Ex Group (*n* = 19)	Control Group (*n* = 19)	*p*-Value	*η* ^2^_p_
AT elongation, mm	3.78 ± 1.27	3.64 ± 0.59	0.012 *	0.17
AT stiffness, N/mm	74.33 ± 13.37	69.38 ± 10.94	0.027 *	0.13
AT strain, %	3.13 ± 0.69	2.78 ± 0.72	0.004 *	0.21
AT stress, N/mm^2^	5.02 ± 1.11	4.43 ± 1.14	0.006 *	0.20
Young’s modulus, N/mm^2^	171.96 ± 66.55	166.32 ± 43.43	0.78	0.002

Abbreviations: PLYO-Ex: plyometric exercises, AT: Achilles tendon. * Significant at *p* < 0.05, *η*^2^_p_: partial eta-squared.

## Data Availability

The data that support the findings of this study are available on reasonable request from the corresponding author.

## Supplementary Materials

The following supporting information can be downloaded at: https://www.mdpi.com/article/10.3390/children9111604/s1, Table S1: Details of the PLYO-Ex program
