# Supplementary material for: Mechanical and Morphological Changes of the Plantar Flexor Musculotendinous Unit in Children with Unilateral Cerebral Palsy Following 12 Weeks of Plyometric Exercise: A Randomized Controlled Trial
Source: Children (Basel). 2022 Oct 22;9(11):1604. doi: 10.3390/children9111604 (PMC9688148; doi:10.3390/children9111604)
Supplement: Supplementary file 1 [file children-09-01604-s001.zip › children-1930214-supplementary.pdf]

**Supplementary Table S1:** Details of the PLYO-Ex program

| Exercise | Characterization | Block 1 | Block 2 | Block 3 |
|---|---|---|---|---|
| Horizontal plyometric exercise-model | | | | |
| Bound | Push off one foot and jump out and forward as far as possible to land on the other foot. | 1 x 5 | 1 x10 | 1 x15 |
| Forward-jump | With forward arm swing, jump as far as possible with both feet | 1 x 5 | 1 x10 | 1 x15 |
| Single-leg forward hop | Hop forward between lines with no stops in both directions | 1 (5RT and 5LT) x 5 | 1 (5RT and 5LT) x 10 | 1 (5RT and 5LT) x 15 |
| Lateral leap | Stand on one leg, stretch out the other leg, then hop laterally | 1 x 5 | 1 x10 | 1 x15 |
| Side-to-side jump | Jump as far as possible with both feet from one side to the other | 1 x 5 | 1 x10 | 1 x15 |
| Vertical plyometric exercise-model | | | | |
| Reciprocal stride-jump | From stride standing, jump up, with feet interchangeably advanced forward between jumps | 1 x 5 | 1 x10 | 1 x15 |
| Squat-jump | Stand with feet shoulder-width apart and squat down, then jump up. On landing, lower the body back into the squat | 1 x 5 | 1 x10 | 1 x15 |
| Tuck-jump | Jump up, approaching the chest with bent knees | 1 x 5 | 1 x10 | 1 x15 |
| High-step hop | Hop up on and down off a 5-inch-high step and switch between both feet | 1 (5RT and 5LT) x 5 | 1 (5RT and 5LT) x 10 | 1 (5RT and 5LT) x 15 |
| High-step jump | Jump up on and down off a 5-inch-high step and switch between both feet | 1 (5RT and 5LT) x 5 | 1 (5RT and 5LT) x 10 | 1 (5RT and 5LT) x 15 |

- Blocks presented as the number of sets/number of repetitions
- Children were asked to perform all repetitions successively with no pauses and encouraged to maximize their efforts as much as possible.
- Rest intervals of 1-2 minutes were allowed between exercise sets.
- A preliminary test of children's performance was conducted in a sample of 5 children to determine the number of repetitions they should start with in the first block.
